# Compatibility Evaluation of Non-Woven Sheet Composite of Silk Fibroin and Polyurethane in the Wet State

**DOI:** 10.3390/polym10080874

**Published:** 2018-08-06

**Authors:** Derya Aytemiz, Yasuhiro Fukuda, Akira Higuchi, Atsushi Asano, Chikako T. Nakazawa, Tsunenori Kameda, Taiyo Yoshioka, Yasumoto Nakazawa

**Affiliations:** 1 Department of Biotechnology and Life Science, Tokyo University of Agriculture and Technology, 2-24-16 Naka-cho, Koganei, Tokyo 184-8588, Japan; g-derya@nms.ac.jp (D.A.); fukuda-yasuhiro@nikke.co.jp (Y.F.); fw4671@go.tuat.ac.jp (A.H.); 2 Department of Applied Chemistry, National Defense Academy, 1-10-20 Hashirimizu, Yokosuka, Kanagawa 239-8686, Japan; asanoa@nda.ac.jp (A.A.); chikakot@nda.ac.jp (C.T.N.); 3 Silk Material Research Unit, Institute of Agrobiological Sciences, National Agriculture and Food Research Organization, Ohwashi, Tsukuba, Ibaraki 305-8634, Japan; kamedat@affrc.go.jp (T.K.); yoshiokat@affrc.go.jp (T.Y.)

**Keywords:** silk fibroin, solid-state NMR, electrospinning, polyurethane

## Abstract

SF/polyurethane composite non-woven sheet was fabricated to evaluate the cardiovascular tissue engineering materials in the wet state. The compatibility and microstructure analyses were carried out on the fabricated SF/polyurethane composite non-woven sheet by thermal analysis and solid-state NMR analysis in the wet state. To evaluate the modulus of elasticity, a tensile test was performed and supported with dynamic viscoelasticity and mechanical analysis. Results showed that SF/polyurethane composites form domains within the non-woven sheet and are in a finely dispersed state while maintaining their structures at a scale of several tens of nm. Moreover, an increase of the loss tangent with low elastic modulus proved that a micromolecular interaction occurs between silk fibroin (SF) and polyurethane molecules.

## 1. Introduction

Cardiac repair patches are sheet-like medical devices that are used for restoring defective areas in the heart caused by congenital diseases, such as ventricular septal defects (VSD) or patent ductus arteriosus (PDA). Current materials used for the reconstruction of cardiac defects include synthetic or xenotypic materials, such as expanded polytetrafluoroethylene (ePTFE) (e.g., Gore-Tex or IMPRA) and glutaraldehyde-fixed bovine pericardium. However, since neither the ePTFE nor heart mucosa after treatment with glutaraldehyde is non-degradable, these cardiac repair patches permanently remain without being absorbed by the body [1]. This may cause thrombosis or calcification in the remote period after surgery and require re-operation. To overcome multiple operations, there are needs of newly-developed cardiac repair patches with high mechanical strength, flexibility, and blood compatibility because it comes in contact with high blood pressure and contractile movement. In addition, it is desirable to have better tissue affinity to prevent calcification, which is a problem of existing products.

Polymers are the largest class of biomaterials used today for scaffold preparation, either in gel or in solid states [2,3,4]. However, the polymeric materials used as tissue-engineering scaffolds in most studies have similarly come from a limited set of polyesters. More recently, investigators have begun exploring a broader array of polymers that may better fit the requirements of particular clinical applications, with emphasis on appropriate mechanical properties, biodegradation rates, drug delivery potential, and cytocompatibility [5,6].

Silk fibroin (SF) is a type of fibrous protein that is the main component of silken threads produced from *Bombyx mori* [7]. SF has a high mechanical strength comparable to that of steel wire by forming a *β*-sheet structure. In addition, the gentle biodegradability and the low inflammation come to attention by some results from in vivo studies [5,6,7,8,9]. Therefore, SF has attracted attention as a tissue engineering material. The degradability and the decomposition behavior in vivo are derived from the secondary structure of SF and the secondary structure of SF can be easily controlled. Therefore, these properties could also be controlled [10,11]. Such utilities and properties make SF a good candidate for regenerative medicine. However, it is difficult to use SF alone as a reconstructive material because of its poor mechanical stability and thrombogenicity.

Polyurethane is a generic name for polymer compounds having urethane bonds formed from polyol and divalent isocyanate. Among them, Pellethane^®^ (PU) is a thermoplastic polyurethane elastomer with excellent in strength and flexibility, and it is characterized by excellent processability. Furthermore, it has an effect of suppressing platelet aggregation in vitro as compared to other polymer compounds, and shows antithrombotic property. Additionally, polyurethane selectively adsorbs proteins mainly composed of albumin in the blood and these prevent recognizing it as a foreign body in vivo. In this way, it is considered that PU is able to give antithrombotic properties to materials [12]. Due to these properties, it has been applied to medical equipment, such as assistive devices for artificial hearts and catheters.

Polymer blends are one of the simple methods having possibilities to provide materials with extended useful properties, such as SF-coated poly(carbonate) urethane membranes, which supported the adhesion and growth of fibroblasts without secreting pro-inflammatory cytokines [13]. Additionally, an in vitro study of SF blended with PU films in various concentrations assessed their capacity to support myoblast differentiation [14]. Many studies have adopted design strategies to create target materials from polymer blends by using electrospinning to ensure cell compatibility of biopolymers and mechanical properties by synthetic polymers [15,16,17]. Although several vascular grafts made of PU or its blends have been developed and evaluated, only a few studies specifically assessed electrospining of PU blends and less for SF/PU blends. In 2016, Yu et al. prepared small-diameter vascular grafts, which possessed a hybrid fibrous structure of an aligned inner layer and a random outer layer. This grafts showed compatible mechanical properties with human coronary artery. Furthermore, endothelial cell culture results exhibited promising cell viability and biocompatibility [18]. In our previous report, we presented that the ordered structures of SF and PU are preserved in the blend sample after electrospining. For SF, the inter- and intra-molecular hydrogen bonds maintained the β-sheet structure, and the interaction was not severely affected by the presence of PU [19]. Further, samples were evaluated by in vivo by implantation into the abdominal aortas of rats, using the ePTFE patch in the control group. At one week after implantation, the SF/PU patch had been infiltrated with cells and collagen fiber. The ePTFE control patch did not accumulate collagen fiber until three months and calcification occurred at 4 weeks. The SF/PU patch did not present any signs of calcification for three months. This study addressed the problems associated with using SF in isolation and showed that the SF/PU patch can be considered as a useful alternative to the ePTFE to overcome the problem of calcification [20]. Additionally, we focused on the effects of different SF concentrations in the SF/PU patch on its biological and physical properties. Three patches of different compositions were created using an electro-spinning method. Each patch type (*n* = 18) was implanted into rat abdominal aorta and histopathology was assessed at 1, 3, and 6 months post-implantation. The results showed that with increasing SF content the tensile strength and elasticity decreased. At six months post-implantation, the higher SF concentration patch demonstrated progressive remodeling, including significantly higher tissue infiltration, elastogenesis and endothelialization compared with lower SF concentration [21]. In addition to rat studies outstanding results also observed with canine descending aorta replacement with a SF/TPU sheet as a patch [22].

In particular, blending biopolymers with synthetic polymers has been reported that the compatibility between the polymers have effect on the properties of the obtained materials [23,24]. Therefore, in the case of development of blend polymer materials, it is important to verify he compatibility of the blended polymers on the molecular level and to consider the influence on the characteristics of the final material. Our previous in vivo studies showed superior characteristics and results compared with commercial sheets. However, the reason why the SF/PU composite non-woven sheets demonstrated good performance in vivo is unknown. The sheets should be exposed to body fluids including blood, and be swollen in water in vivo. Therefore, in order to understand the properties of biomaterials realistically, the materials should be evaluated in wet state, similar to intravital environment.

Evaluations of compatibility and microstructure of the fabricated SF/ PU composite non-woven sheets were carried out by thermal analysis and solid-state NMR analysis in dry and wet state. The mechanical properties of SF/ PU composite non-woven sheets were evaluated with tensile test and dynamic viscoelasticity measurement.

## 2. Materials and Methods

### 2.1. Production of Silk Fibroin Sponge

Silk fibers were obtained from *Bombyx mori* cocoons and degummed as previously described [25]. Briefly, silk fibers were obtained by reeling and weaving silk cocoons in boiled water at 80 °C for about 15 min. The dried silk threads were placed in a mixture of sodium carbonate (0.08%, *w*/*v*) and Marseille soap (0.12%, *w*/*v*) at 95 °C for 120 min. This process was repeated to completely remove the silk sericin from the raw silk fibers. The removal of silk sericin was confirmed using a SEM (VE-7800, Keyence Co., Osaka, Japan). Degummed SF fibers were dissolved in 9 M lithium bromide (LiBr) solution at a concentration of 10% (*w*/*v*) at 37 °C for 3 h. Then this solution was dialyzed against distilled water until LiBr was completely removed. The SF aqueous solution was obtained by purification of the dialyzed solution by centrifugation. The final concentration of the SF aqueous solution was approximately 4% (*w*/*v*). To obtain SF sponge, SF aqueous solution was diluted to 1% (*w*/*v*) and then freeze-dried over 24 h.

### 2.2. Fabrication of SF/PU Composite Non-Woven Sheet

**Figure 1:**
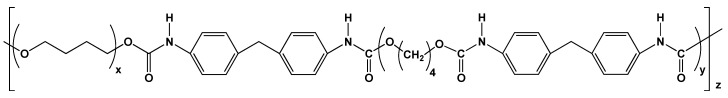
Structure of the hard segment of Pellethane^®^. *M*n = 80,000, *M*w = 160,000.

SF sponge and PU (Pellethane^®^ 2363-80AE, The Lubrizol Co. Ltd., Wickliffe, OH, USA) were used to prepare SF/PU composite patches. The obtained SF sponge and PU were dissolved in 1,1,1,3,3,3-hexafluoro-2-propanol (HFIP) and then blended at 6 (*w*/*v*, %). Figure 1 shows the chemical structure of the Pellethane^®^ molecule.

After that, SF/ PU HFIP solutions were filled in syringe attached to a needle and electrospun for 3 h with a high-voltage power supply under laboratory condition (Esprayer ES-2000S, Fuence Co. Ltd., Tokyo, Japan). The distance between electrodes was fixed at 10 cm. The composition of SF sponge/PU samples changed from 10/0, 7/3, 5/5, 3/7, and 0/10. The conditions of the high-voltage power supply was set-up from 15 kV to 20 kV to obtain the best spinning process for each sample individually: electrospinning conditions of each sample are shown in Supplementary Table S1. The applied voltage was tuned to suppress the dispersion of the fiber diameter in each composite patch and an aluminum plate of 50 × 50 × 1 mm was used as a collector. The discharge rate of solutions and spinning distance were 12 µL/min and 10 cm respectively. The fabricated sheets were dried overnight and placed in 100% relative humidity for 24 h at 37 °C atmosphere to insolubilize SF in the composites. Then, the sheets were immersed in water for 72 h to remove HFIP and were dried again. For wet state measurements, the sheets were immersed in water for 24 h. The percentage of water content was calculated by the formula: (wet weight—dry weight)/wet weight) × 100.

### 2.3. Confirmation of Fabrication and Morphological Evaluation of SF/PU Composite Non-Woven Sheet by SEM Observation

A porous structure is desirable for cell and tissue immigration and the growth of tissue engineering materials because the extracellular matrix (ECM) of native tissue has a similar structure [19]. The electrospun SF/PU composite patches were evaluated by gross observation and their morphology was characterized using a SEM operated at 10 kV with a 1000× magnification (VE-7800 Keyence Co., Osaka, Japan). The obtained image was analyzed and the fiber diameter was determined by randomly selecting 50 fibers from each image.

### 2.4. High-Resolution Solid-State ^13^C NMR Measurement

All of the solid-state NMR measurements were performed using a Varian NMR system 400 WB spectrometer (Agilent Technologies, Santana Clara, CA, USA) with an operating frequency of 100.57 MHz for ^13^C using 6.0-mm rotor. The magic-angle spinning (MAS) rate was set to 7 kHz and the TPPM ^1^H-decoupling method [26] was used. The ^13^C chemical shifts were measured relative to tetramethylsilane (TMS) using the methylene carbon signal at 29.47 ppm for solid adamantine as an external standard. The ^13^C spectra were obtained by the combined use of cross polarization (CP) and MAS. The contact time of CP was 1 ms. Proton spin-lattice relaxation times in the laboratory frame (*T*_1_^H^) were indirectly measured from the well-resolved ^13^C signals enhanced by CP of 1 ms applied after the inversion-recovery pulse sequence for ^1^H [19].

### 2.5. Mechanical Property Test

The mechanical properties of non-woven SF/PU composite sheets were evaluated with a tensile test and dynamic viscoelasticity measurement. An EZ-test machine (Shimadzu Seisakusho Co., Ltd., Kyoto, Japan) was used to evaluate tensile of wet state non-woven specimens. The dimensions of the sample piece were having a width × grip length of 3 × 15 mm. Measurement was carried out to the point where the elongation reached at 200% of those under 2 mm/min tensile speed. The modulus of elasticity was calculated from the elongation 1–2% section of the obtained stress-strain curve (*n* = 3).

Dynamic viscoelasticity and dynamic mechanical analysis (DMA) measurements were performed using DVA-205 (IT measurement regulation, Co., Ltd., Osaka, Japan). Dynamic viscoelasticity measurements observed in tensile mode and at a frequency of 1 Hz. Measurement was carried out continuously at 37 °C water temperature and the average value of the storage elastic modulus (*E*’), loss elastic modulus (*E*″), and loss tangent (tan *δ*) evaluated from the results obtained after 10 min. For wet state evaluation a water bath was attached to the measurement equipment. To perform a dynamic mechanical analysis measurement, the specimens were heated from −150 °C to 300 °C with a heating rate of 2 °C/min. Analysis was performed under 0.08% tensile strain and at 1, 4, 10, 16, and 64 Hz frequencies. Measurement was carried out in tensile mode and the specimen was processed so as to have a width × grip length of 3 × 15 mm. The values of the glass transition (*T*_g_) were read off as the temperatures of the peak of the loss factor.

## 3. Results

### 3.1. Morphology of SF/PU Composite Non-Woven Sheets

**Figure 2:**
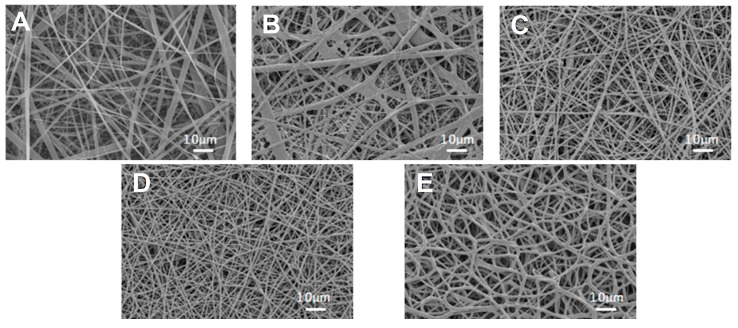
SEM image of SF/PU = (**A**) 10/0; (**B**) 7/3; (**C**) 5/5; (**D**) 3/7; and (**E**) 0/10 composite non-woven sheet.

Figure 2 shows the SEM images of SF/PU composite non-woven sheets. All sheets were composed of randomly oriented micro- and nano-scale fibers. Thus, it appears that mixing of SF and PU did not inhibit the spinning of ultrafine fibers. The average fiber diameters of composite non-woven sheets are shown in Table 1. The averages were evaluated around 1 μm for each sample. However, the fiber diameters of pure SF non-woven sheet showed obviously wider variation than the others (both from Figure 2 and Table 1). Fiber diameter and the variation became smaller with the increase of PU concentration in the composite sheets. In particular, SF/PU = 3/7 composite non-woven sheet was composed of the finest fibers with the narrowest variation in the diameter. The electrospinning process succeeded in making SF/PU composite non-woven sheets having pores or retiform structure mimicking to the structure of native ECM. It is desirable structure for tissue engineering materials because it is a favorable structure for cells and tissues to immigrate and to grow.

### 3.2. Structure Analysis by Solid State NMR Measurement

**Figure 3:**
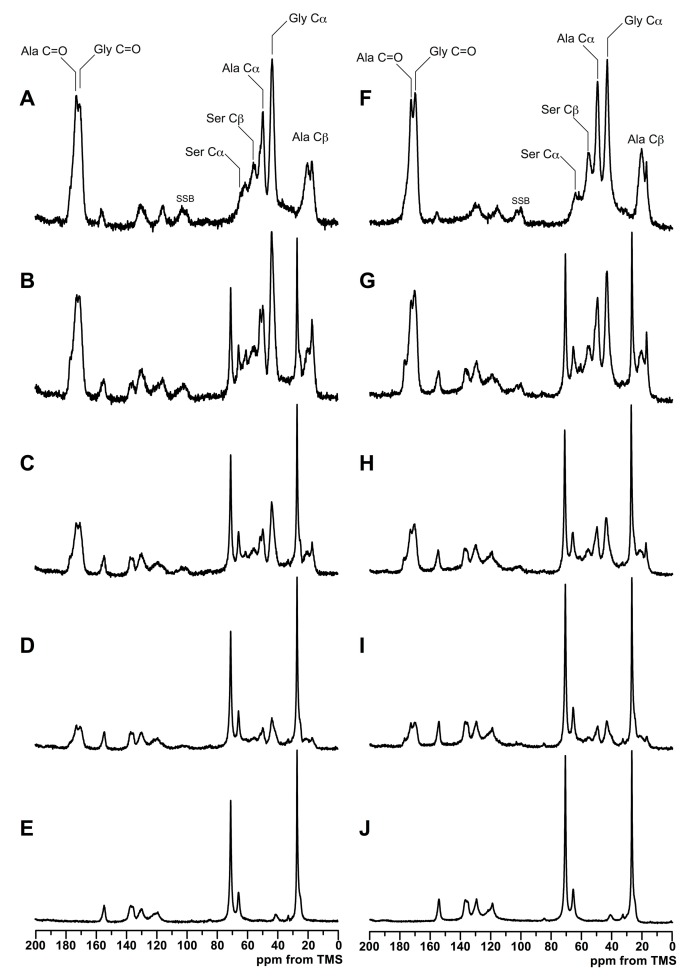
^13^C CP/MAS NMR spectra of non-woven sheets in dry (**A**–**E**) and wet (**F**–**J**) samples. SF/PU = 10/0 (**A**,**F**); 7/3 (**B**,**G**); 5/5 (**C**,**H**); 3/7 (**D**,**I**); and 0/10 (**E**,**J**); respectively.

**Figure 4:**
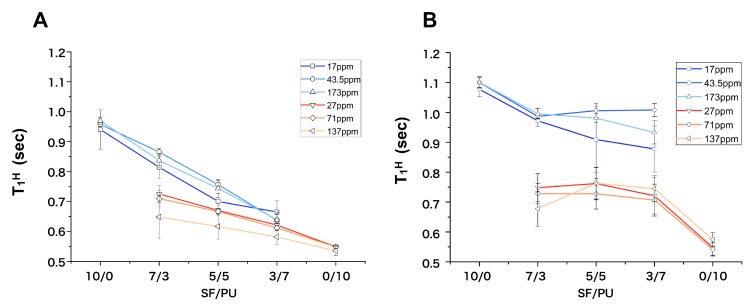
*T*_1_^H^ plot of SF (C=O, 173 ppm; Gly C*α*, 43.5 ppm; Ala C*β*, 17 ppm) and PU (phenyl groups, 137 ppm; CH_2_, 71.0 ppm; CH_2_, 27.0 ppm) in SF/PU composite non-woven sheets in dry (**A**) and the wet state (**B**).

To compare the structural characteristics of SF/PU non-woven sheets between pure and composite sheets, the ^13^C CP/MAS NMR spectra shown in Figure 3, and the proton spin-lattice relaxation time (*T*_1_^H^), shown in Figure 4, were measured because the polymer interaction frequently affects solid-state NMR spectrum [27]. Moreover, the spectra and *T*_1_^H^ values in dry and wet states were also compared because the characterization in wet state was important in view of the actual condition which the sheets were usually used as a scaffold in living animal [28]. The ^13^C CP/MAS NMR spectra of SF/PU composite sheets in the dry and the wet states are shown in the left and the right column of Figure 3, respectively. The dry state ^13^C CP/MAS NMR spectrum of pure SF non-woven sheet (Figure 3A) showed the typical spectrum pattern of silk II structure [29]. For example, the characteristic peaks derived from the random-coil and the repeated *β*-turn structure at 16.6 ppm and the *β*-sheet structures at 19.6 ppm were observed in Ala C*β* region. In addition, all of the other peaks, that is, Gly C*α* peak at 42.5 ppm, Ala C*α* peak at 49.0 ppm, Gly C=O peak at 169.9 ppm and Ala C=O peak at 173.0 ppm indicate that the main structure of this sheet is the *β*-sheet. Small shoulder peaks of C=O at 176 ppm assigned to Ala C=O in repeated *β*-turn structure were observed in all of the spectra. This is agreeing with the fact that the peaks at 16.6 ppm assigned to repeated *β*-turn structure are sharper than typical Ala C*β* peak pattern of random coil. Therefore, pure SF sheet in the dry state forms silk II structure composed of *β*-sheet structure mainly, although it includes silk I structure a little. The spectrum of pure PU in the dry state, in Figure 3E, showed the typical polyurethane peaks: the soft segments of CH_2_ and O–CH_2_ unit are around 27.0 and 71.0 ppm and the hard segments mainly consisted of phenyl groups are 100–160 ppm. The chemical shift values of all peaks derived from both SF were not changed even though mixing with PU at any ratio as shown in Figure 3B–D, the weight ratio of SF/PU = 7/3, 5/5, 3/7, respectively. This fact shows those of characteristic structures of SF were unchanged even in the SF/PU composite sheets. These observation supports that the SF chains are not in close proximity to the PU chains on an atomic level to affect the strength of their electron densities: for example, no hydrogen bonding interaction between SF and PU was detected.

Focusing on the changes in the proportion of the constituent of Ala C*β* peaks, the proportion of the peak at 16.6 ppm assigned to random coil and *β*-turn was decreased, and that of the peaks assigned to *β*-sheet structure, at 21.9 ppm in particular, was increased instead, with increasing of the PU ratio in the dry state without SF sheet (Table 2). The different behavior of pure SF in the proportion of *β*-sheet structure might be the influence of the CH_2_ peak derived from PU existing at around 27.0 ppm, except the pure SF spectrum, while the influence has been removed carefully at the deconvolution. In the wet state, there is no drastic change in ^13^C CP/MAS NMR spectrum compared to that in the dry state, although the shape of Ala C*α* peak around 52 ppm was a bit different. This difference considered to be caused from the difference of the *β*-sheet content, and is according to the deconvolutions of Ala C*β* peak (Table 2). Therefore, main structures of both SF and PU were not different between dry and wet state, except the proportion of the *β*-sheet structure in SF chains. Considering the characteristics of the ^13^C CP/MAS NMR method, however, these differences could have been caused not only by the structural difference but also by the effect of the motional differences between *β*-sheet structure and random coil in the wet state. That is, the part of SF in comparably flexible random coil state would be mobile in the wet state and become less efficient at cross polarization, then the proportion of the peaks assigned to *β*-sheet was relatively increased. From these results, it is considered that the proximity between SF and PU molecules are compatible enough to not inhibit secondary structure formation of SF.

Figure 4 shows the proton spin-lattice relaxation times (*T*_1_^H^) against the SF/PU composition in both dry (A) and wet (B) states. When the fast ^1^H spin diffusion occurs between SF and PU domains, the different spin energies of them are averaged out and become identical. Such phenomenon occurs in the case that both polymers are in close proximity within several tens nm [19]. The *T*_1_^H^ values were obtained from peaks ascribed to Ala C=O at 173 ppm, Gly C*α* at 43.5 ppm, and Ala C*β* at 16.6 ppm for SF and for PU, the phenyl groups at 137 ppm, and CH_2_ peaks at 71.0 ppm and at 27.0 ppm. In the dry state, the *T*_1_^H^ values of SF gradually decreased from 0.9 s for pure SF down to 0.65 s for SF/PU = 3/7 with decrease of SF composition. On the other hand, those of PU increased from 0.55 s for pure PU to 0.7 s for SF/PU = 7/3 with increase of SF composition (Figure 4A). The *T*_1_^H^ values of SF were very close to those of PU in the composites, especially in 5/5 and 3/7 ratios. This observation shows that the ^1^H spin diffusion occurred significantly between SF and PU chains. In the solid-state NMR measurements, the ^1^H spin diffusion phenomenon originally occurred in a polymer: the effect is limited in several tens nm scale. If the SF and PU chains are in close proximity on a scale of several tens nm, both *T*_1_^H^ values observed in the composites will be in agreement with each other because of effective ^1^H spin diffusion. Therefore, the observation shown in Figure 4A suggested that SF and PU chains in the composite non-woven sheets are sufficiently miscible with each other. This observation suggested the model which both SF and PU molecules formed small domains with in several tens nm in diameter and their domains were dispersed each other, because it is indicated that the original structure of SF and PU were maintained in the composite sheets from the ^13^C CP/MAS NMR spectra (Figure 3). In the wet state, the *T*_1_^H^ values of SF were longer than that in dry state, although that of PU were almost same in dry and wet state. Therefore, water molecules were suggested to be interacted with SF molecules mainly. It is supported from the water contents shown in Table 3. The water contents of the composite sheets were decreased with increase of PU ratio and that of pure PU sheet was clearly lower than that of the others which included SF molecules. In the composite sheets, the *T*_1_^H^ values of SF were not in agreement with those of PU, even though both values changed towards to mutual values (Figure 4B). Namely, the *T*_1_^H^ values of SF decreased from 1.1 s down to 0.95 s and those of PU increased from 0.55 s to 0.75 s. It indicates an existence of a weak ^1^H spin diffusion. The ^1^H spin diffusion is effective in the rigid state, so that the observation in Figure 4B indicated that the spin diffusion was weakened by relatively fast movement of SF chains and/or the mediacy of water molecules in the wet condition. That is, the different proxemics degree of *T*_1_^H^ values between the dry and wet states is considered not to be the change of the model or the domain size, but to be the change of the spin diffusion way.

Therefore, from these results, it is presumed that within the non-woven sheet, SF molecules and PU molecules aggregate to some degree to form domains.

### 3.3. Mechanical Properties of SF/PU Composite Non-Woven Sheets

**Figure 5:**
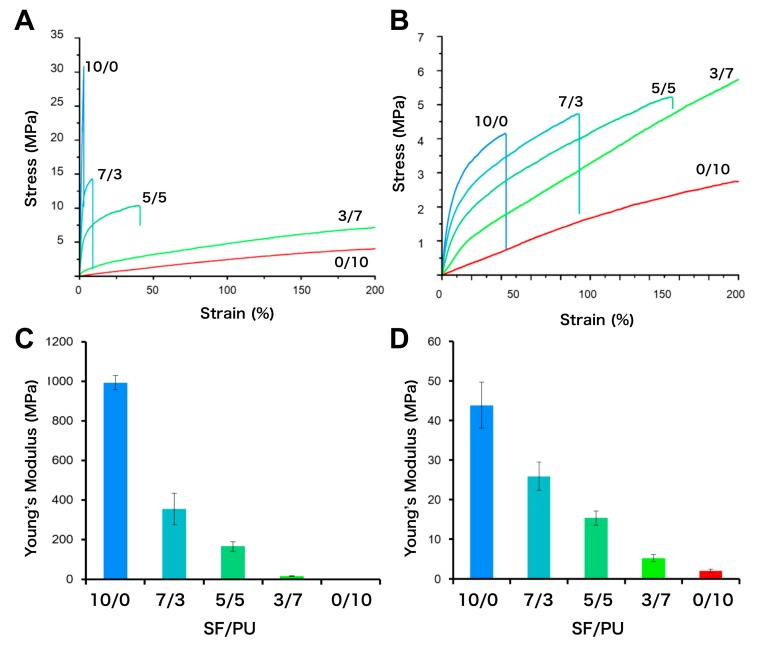
Stress-strain curve of SF/PU composite non-woven sheets in dry (**A**) and wet (**B**) states; and the Young’s modulus calculated from stress-strain curves in dry (**C**) and wet (**D**) state. The Young’s modulus of SF/PU = 0/10 in the dry state (**C**) could not be shown in the bar because of the very low value (2.9 ± 0.1 MPa) compared to the others.

Figure 5 shows the stress-strain curves (A and B) and the Young’s modulus (C and D) of SF/PU composite non-woven sheets in dry (A and C) and wet (B and D) state. It is clear from the Figure 5A,B that the breaking strains of SF/PU composite non-woven sheets increased with increase of PU content. Although for those of PU-rich sheets, SF/PU = 3/7 and 0/10, breaking were not observed because of the higher breaking strain of more than 200%. As compared with same SF/PU ratio sheets in dry and wet states, the breaking strain in dry state were lower than that of wet states. The breaking stresses of those sheets were decreased with increase of PU content in dry state, those in the wet state were increased conversely. In the same ratio sheets, the breaking stress in dry state were higher than that of wet states. The Young’s moduli were decreased with increase of PU content in both dry and wet states. The Young’s moduli in wet states were predominantly lower than that of same ratio sheets in dry state. Focusing on pure non-woven sheets, in the case of SF, i.e., 10/0 sheet, the breaking stress was decreased and the breaking strain was increased by immersing it in water, and then the Young’s modulus was predominantly decreased as a result from 1000 MPa in dry state to 40 MPa in wet state. It is known that wet SF fiber is more elastic compare to dry fiber [28]. The same tendency was observed in non-woven sheets even after blending SF with PU. On the other hand, there was no significant difference of the breaking stress, breaking strain and Young’s modulus of pure PU sheet in between dry and wet state. On the basis of water content, it is considered that the mobility and/or network such as hydrogen bonding of SF molecules were affected by water and it changed the properties of composite non-woven sheets as a result. The Young’s moduli of composite non-woven sheets were decreased by increase of PU content even in wet state which that of pure SF was drastically reduced, although the decreasing behavior was not linear. This was because the Young’s modulus of pure PU was lower than that of pure SF in the wet state. That is, the lower Young's modulus of PU was maintained in the composite sheets and resulted in PU’s proportion-dependent lower Young’s modulus of the composite sheets. This result supports the model described from the NMR data, in that small domains of SF and PU are dispersed within each other.

In the view of application to a material for the circulatory system, the Young’s modulus values of several tens MPa represented by SF/PU = 3/7 in dry state and by 5/5 and 3/7 in the wet state were comparable to that of ePTFE which is commercial used cardiac patch material and represents the Young’s modulus value of about 20 MPa in dry state and 15 MPa in the wet state. That is, SF/PU composite non-woven sheets prepared in the proportion of SF/PU = 5/5 to 3/7 would be a good material to repair the circulatory system, such as the heart and vasculature, since the materials should be exposed to water in vivo.

**Figure 6:**
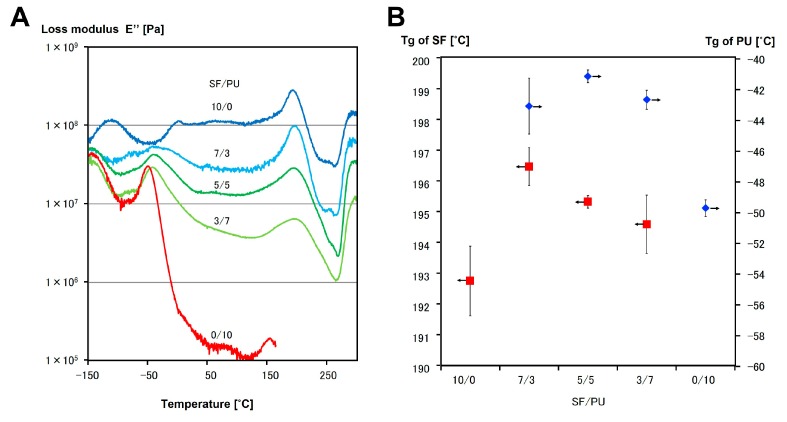
The loss modulus (*E*″) (**A**), and the glass transition point (*T*_g_) read from *E*″ curve (**B**) of SF/PU = 10/0, 7/3, 5/5, 3/7 and 0/10.

It is known that a loss elastic modulus peak obtained from DMA measurement shows the glass transition point (*T*_g_). The loss modulus (*E*″) of SF/PU = 10/0, 7/3, 5/5, 3/7, and 0/10 non-woven sheets were shown in Figure 6A. It is known that the peak at around −50 °C and 200 °C are assigned to the *T*_g_ of PU and SF, respectively. In here, the peak at 193 °C was observed in the SF curve, whereas the peak at −50 °C was observed in the PU curve. These two peaks were also observed in the curves of all composite non-woven sheets, SF/PU = 7/3, 5/5, and 3/7. The *T*_g_ values read from the curves were shown in Figure 6B. The *T*_g_ values of composite non-woven sheets derived from SF were decreased with increase of the proportion of PU, although the values were higher than that of pure SF non-woven sheet. The *T*_g_ values of PU in composite non-woven sheets were higher than that of pure PU, and it tended to decrease with increase of the PU ratio. It is considered that the fact of both *T*_g_ were decreased with increase of PU ratio in composite non-woven sheets was due to a difference of the thermal conductivity. That is, the thermal conductivity of PU might be higher than that of SF. However, the other facts that both *T*_g_s of composite non-woven sheets were clearly observed and were different from pure ones indicated that both of the crystals of SF and PU were maintained even after mixing together, and were interacted with each other. The other peaks on SF curve at around −100 °C and 0 °C were derived from adsorbed water. These peaks were not observed in other curves. In the curve of PU, a peak at around −140 °C derived from the rotational relaxation of benzene-rings was obtained and a meltdown was caused at around 160 °C. Such a meltdown was not observed in composite non-woven sheets, even after being heated to 300 °C. It was indicated that the thermal resistance of PU was improved drastically by mixing with SF.

The measurement of SF/PU = 0/10 non-woven sheet was stopped at around 150 °C due to melting of the sheet. However, melting was not observed in SF/PU = 7/3, 5/5, and 3/7 non-woven sheet. Therefore, it was confirmed that the thermal stability of the non-woven sheets dramatically improved by SF blending.

## 4. Conclusions

In this study, as a cardiovascular tissue engineering materials, SF/PU composite non-woven sheets with different ratio were created by using the electrospinning method and were evaluated the miscibility and the mechanical property.

The ^13^C CP/MAS NMR spectra for fabricated sheets showed the characteristic peaks of the Ala C*β* and the C=O formed *β*-turn and *β*-sheet structures in SF, and the typical PU peaks, CH_2_, O–CH_2_, and benzene ring. It is considered that the proximity between SF and PU molecules are compatible enough to not inhibit secondary structure formation of SF. In the wet state, the motility of *β*-turn structure in SF/PU composite non-woven sheets was increased compared to that in the dry state, as shown by the sharpness of peaks for random coil and *β*-turn structures. From the *T*_1_^H^ analyses, it is confirmed that SF and PU were miscible in several tens of nm, because the values of the peaks derived from SF and PU in the composite non-woven sheets came close with each other. However, the *T*_1_^H^ values in the wet state did not come closer than that in the dry state. It is suggested that water molecules intermediate the interaction between SF and PU domains. From the stress-strain curves, it was revealed that the values of tensile stress and the Young’s modulus were increased, whereas the values of strain were decreased with the increase of the SF ratio. To compare the dry and wet states, the values of tensile structure and Young’s modulus in wet states were significantly lower than that in the dry state. Additionally, DMA results indicated that the crystalline components of both SF and PU were preserved in the composite non-woven sheets.

From the above results, we succeeded in creation of SF/PU non-woven sheets as a candidate material for soft tissue and in evaluating their microstructure in dry and wet states. These results are expected to be useful knowledge for designing new tissue engineering materials for the circulatory system.

## Figures and Tables

**Table 1:** Fiber diameters of SF/PU composite non-woven sheets.

SF/PU	10/0	7/3	5/5	3/7	0/10
Fiber diameter (µm)	1.2 ± 1.1	0.9 ± 0.6	0.9 ± 0.4	0.7 ± 0.2	1.2 ± 0.4

**Table 2:** Fraction of the random coil and the *β*-turn structure (16.6 ppm) and the *β*-sheet structure (19.6 ppm and 21.9 ppm) of SF/PU composite sheets. Determined by the deconvolution of the Ala C*β* peaks in wet and dry state.

Chemical Shifts of Ala C*β* Peak in SF (Structure Which Was Assigned to)	Dry State (%)				Wet State (%)			
	SF/PU Ratio				SF/PU Ratio			
	10/0	7/3	5/5	3/7	10/0	7/3	5/5	3/7
16.6 ppm (random coil and repeated *β*-turn)	47	52	42	35	32	36	38	27
19.6 ppm (*β*-sheet)	27	36	42	34	57	52	34	63
21.9 ppm (*β*-sheet)	26	12	17	31	12	12	28	10

**Table 3:** Percentage water content of non-woven sheets.

SF/PU	10/0	7/3	5/5	3/7	0/10
Water content (%)	73 ± 1.7	71 ± 1.4	70 ± 0.7	65 ± 0.5	16 ± 6.3

## Supplementary Materials

The following is available online at http://www.mdpi.com/2073-4360/10/8/874/s1, Table S1. Electrospininng conditions for each sample.
