# Supplementary material for: Compatibility Evaluation of Non-Woven Sheet Composite of Silk Fibroin and Polyurethane in the Wet State
Source: Polymers (Basel). 2018 Aug 6;10(8):874. doi: 10.3390/polym10080874 (PMC6403721; doi:10.3390/polym10080874)
Supplement: Supplementary file 1 [file polymers-10-00874-s001.pdf]

Compatibility Evaluation of Non-Woven Sheet Composite of Silk Fibroin and Polyurethane in the Wet State

Derya Aytemiz, Yasuhiro Fukuda, Akira Higuchi, Atsushi Asano, Chikako T. Nakazawa, Tsunenori Kameda, Taiyo Yoshioka and Yasumoto Nakazawa

Table S1. Electrospininng conditions for each sample.

Sample name	Component ratio (%)		Voltage	Injections speed
	SF	PU (Pellethane®)		
SF/PU = 10/0	100	0	17 kV	12 μl/min
SF/PU = 7/3	70	30	17 kV	12 μl/min
SF/PU = 5/5	50	50	16 kV	12 μl/min
SF/PU = 3/7	30	70	15 kV	12 μl/min
SF/PU = 0/10	0	100	20 kV	12 μl/min
